# Time-Frequency Domain Analysis of Quantitative Electroencephalography as a Biomarker for Dementia

**DOI:** 10.3390/diagnostics15121509

**Published:** 2025-06-13

**Authors:** Chanda Simfukwe, Seong Soo A. An, Young Chul Youn

**Affiliations:** 1 Department of Bionano Technology, Gachon University, Seongnam-si 1342, Republic of Korea; chandaelizabeth94@gmail.com; 2 Department of Neurology, College of Medicine, Chung-Ang University, Seoul 06974, Republic of Korea; 3 Department of Medical Informatics, Chung-Ang University College of Medicine, Seoul 06974, Republic of Korea

**Keywords:** Alzheimer’s disease, biomarkers, quantitative electroencephalography, frequency domain analysis

## Abstract

Biomarkers currently used to diagnose dementia, including Alzheimer’s disease (AD), primarily detect molecular and structural brain changes associated with the condition’s pathology. Although these markers are pivotal in detecting disease-specific neuropathological hallmarks, their association with the clinical manifestations of dementia frequently remains poorly defined and exhibits considerable variability. These biomarkers may show abnormalities in cognitively healthy individuals and frequently fail to accurately represent the severity of cognitive and functional impairments in individuals with dementia. Research indicates that synaptic degeneration and functional impairment occur early in the progression of AD and exhibit the strongest correlation with clinical symptoms. This identifies brain functional impairment measurements as promising early indicators for AD detection. Electroencephalography (EEG), a non-invasive and cost-effective method with high temporal resolution, is used as a biomarker for the early detection and diagnosis of AD through frequency-domain analysis of quantitative EEG (qEEG). Many researchers demonstrate that qEEG measures effectively identify disruptions in neuronal activity, including alterations in activity patterns, topographical distribution, and synchronization. Specific findings along the stages of AD include impaired neuronal synchronization, generalized EEG slowing, and an increase in lower-frequency bands accompanied by a decrease in higher-frequency bands of resting state EEG. Moreover, qEEG helps clinicians effectively correlate indicators of AD neuropathology and distinguish between various forms of dementia, positioning it as a promising, low-cost, non-invasive biomarker for dementia. However, additional clinical investigation is required to clarify the diagnostic and prognostic significance of qEEG measurements as early functional markers for AD. This narrative review examines time-frequency domain qEEG analysis as a potential biomarker across various types of dementia. Through a structured search of PubMed and Scopus, we identified studies assessing spectral and connectivity-based qEEG features. Consistent findings include EEG slowing, reduced functional connectivity, and network desynchronization. The review outlines key methodological challenges, such as lack of standardization and limited longitudinal validation, and recommends integrative, multimodal approaches to enhance diagnostic precision and clinical applicability.

## 1. Introduction

Electroencephalography (EEG), a non-invasive and cost-effective technique with high temporal resolution, is increasingly utilized as a functional biomarker for detecting synaptic dysfunction across the dementia spectrum from early subjective cognitive decline (SCD) and mild cognitive impairment (MCI) to clinically diagnosed Alzheimer’s disease (AD) through quantitative EEG (qEEG) analysis [1,2,3]. The qEEG involves the numerical analysis of EEG data, often presented in a color-coded format on a single summary page [4,5]. This approach enables neurologists to interpret EEG data more efficiently than traditional EEG methods. AD is the leading cause of dementia, characterized by a progressive decline in cognitive abilities and memory, predominantly affecting individuals aged 65 and older [6,7]. The hallmark pathological features of AD include the buildup of amyloid β (Aβ) peptides, forming senile plaques, and neurofibrillary tangles made of hyperphosphorylated tau proteins [8]. These pathological alterations result in neuronal death and synaptic dysfunction, particularly in the hippocampus and cortical regions essential for cognitive processes [9,10,11]. Consequently, these changes lead to neuronal activity disruptions commonly observed in patients with AD.

The global prevalence of AD is expected to double over the next two decades due to rising life expectancy in many populations [12]. The dementia spectrum includes SCD, a self-perceived worsening of memory without objective impairment on neuropsychological tests as a preclinical stage, followed by MCI, where cognitive deficits are measurable but do not interfere with daily activities [2,13]. In contrast, Alzheimer’s disease (AD) involves progressive cognitive deterioration that ultimately disrupts independent functioning [14,15]. SCD and MCI are considered high-risk states for progression to AD [13,14,15]. Despite massive research efforts and substantial financial investments in dementia drug discovery over the past decade, the complexity of AD pathophysiology has hindered the development of effective treatments, highlighting the urgent need for novel therapeutic approaches [16]. Moreover, various degrees of cognitive impairment might be present in people with similar AD-related neuropathological changes, such as brain atrophy and amyloid accumulation, and vice versa. This phenomenon results from individual variability in resistance to AD-related disease and is known as brain and cognitive reserve [17]. These findings highlight the necessity for new biomarkers to clarify individual vulnerability to the biological substrates of AD, disease pathology, and clinical symptoms.

Although many established biomarkers focus on advanced neuropathological changes such as brain atrophy or amyloid accumulation, qEEG offers the potential to identify synaptic dysfunction at preclinical stages, including SCD and MCI [18]. Several studies have shown that alterations in EEG oscillatory activity, such as increased frontal theta power, reduced posterior alpha activity, and decreased functional connectivity, can be detected in these early phases, often preceding detectable changes on structural magnetic resonance imaging (MRI) or in cerebrospinal fluid (CSF) biomarkers [19,20]. These qEEG patterns have also been associated with early behavioral symptoms, including impairments in attention, executive function, and memory processing [21,22]. This early detection capacity offers significant clinical advantages for timely diagnosis, monitoring of disease progression, and stratifying patients for early therapeutic intervention.

To support this narrative review, we conducted a structured literature search using PubMed (pubmed.ncbi.nlm.nih.gov) and Scopus (www.elsevier.com), focusing on studies that explored time-frequency domain qEEG features in dementia. Search terms included combinations such as “qEEG biomarkers”, “EEG spectral power in Alzheimer’s disease”, “frontal alpha asymmetry”, “EEG functional connectivity in dementia”, “entropy and complexity in EEG”, “EEG synchronization likelihood”, “global field power in MCI”, “cross-entropy EEG analysis”, and “EEG-based machine learning in dementia subtypes”. These terms were selected to encompass a broad range of neurophysiological measures and clinical populations, including AD, MCI, SCD, frontotemporal dementia (FTD), vascular dementia (VaD), and dementia with Lewy bodies (DLB).

This review investigates the potential of frequency-domain qEEG analysis as a noninvasive, early marker of synaptic impairment in AD. Unlike previous reviews that have primarily focused on classical power spectral analysis in AD, this study uniquely synthesizes time-frequency domain qEEG features, including spectral complexity, connectivity, and entropy-based metrics across various dementia subtypes. It also highlights critical methodological gaps, such as inconsistent EEG preprocessing protocols and limited control for confounding variables, which have not been systematically addressed in prior work. This review is based solely on previously published studies and does not include any original research involving human or animal subjects.

## 2. Synaptic Dysfunction in Alzheimer’s Pathology

Synaptic dysfunction is a hallmark of AD and plays a central role in its pathogenesis and progression [23]. In AD, the loss of synaptic integrity disrupts neuronal communication, leading to cognitive decline and memory impairments [24]. Key contributors to synaptic dysfunction include the accumulation of Aβ oligomers, which impair synaptic transmission, and tau protein hyperphosphorylation, destabilizing cytoskeletal structures essential for synaptic health [8,9,10]. These pathological processes result in reduced synaptic plasticity, impaired long-term potentiation (LTP), and eventual synapse loss, particularly in brain regions critical for memory and cognition, such as the hippocampus and cortex [9,10].

Recent research indicates synaptic disruption is more strongly linked to cognitive decline than conventional AD markers like amyloid plaques and neurofibrillary tangles [9,25,26]. This emphasizes the need to prioritize synaptic health in early-stage interventions and investigate biomarkers like qEEG to detect synaptic impairments [2,3,4,5]. Gaining a deeper understanding of the mechanisms driving synaptic dysfunction could provide critical insights for developing innovative therapeutic approaches to preserve cognitive function in individuals with AD.

## 3. EEG-Based Synaptic Markers in AD

The EEG records the functional activity of brain synapses in real-time. The summated excitatory and inhibitory postsynaptic potentials generated by groups of pyramidal neurons in the brain’s gray matter are the main sources of the oscillatory signals picked up by EEG at the scalp. These pyramidal neurons produce detectable current flows as scalp potentials through EEG because they are arranged spatially and perpendicular to the cortical surface [27]. Neural network activity during normal and pathological aging is commonly studied using the resting-state EEG (rEEG), usually recorded under eyes-closed resting (ECR) settings. This is because it enables brain activity measurement while participants are awake but not executing specific tasks [27,28].

Additionally, neuroimaging techniques are crucial for the early detection of AD, helping to reduce misdiagnosis, enhance disease management, and facilitate faster, more effective treatment options [29]. However, advanced imaging modalities such as positron emission tomography (PET) and MRI, while comprehensive, have notable limitations, including high costs, time-consuming procedures, lack of mobility, and risks associated with radiation exposure [30]. EEG, by contrast, holds great potential to address these challenges and serve as a valuable tool in diagnosing AD [31].

Emerging studies suggest that qEEG can detect early neural alterations even before the onset of measurable cognitive decline [18,19]. In individuals with SCD and early MCI, qEEG has revealed specific abnormalities such as reduced alpha power and increased frontal and parietal theta activity [31,32]. These patterns have been consistently linked to early impairments in attention, executive function, and working memory domains commonly affected in the initial phases of neurodegeneration [18]. Crucially, such functional disruptions may be captured by qEEG before detectable structural changes appear on MRI or abnormalities arise in cerebrospinal fluid biomarkers [32].

Magnetoencephalography (MEG) parallels EEG’s methodological foundations, detecting magnetic fields orthogonal to neuronal electrical currents. However, MEG is currently primarily utilized in research settings [33,34]. Meanwhile, transcranial magnetic stimulation (TMS) has emerged as a non-invasive neurophysiological modality with promising utility in evaluating synaptic integrity in AD through its capacity to probe deficits in long-term potentiation (LTP) and cortical plasticity mechanisms [35]. However, its application as a diagnostic biomarker for dementia in clinical and research contexts lacks robust validation. Concurrently, substantial advancements have focused on in vivo synaptic imaging methodologies, notably synaptic vesicle glycoprotein 2A (SV2A) PET, which provides spatially resolved quantification of synaptic density and degeneration patterns [36]. Although reductions in SV2A expression have been consistently documented in AD-affected brain tissue [36], the precise pathophysiological role of this protein, its mechanistic involvement in disease progression, and the pharmacokinetic properties of existing SV2A-targeted radioligands remain incompletely characterized and require further investigation [35].

**Figure 1:**
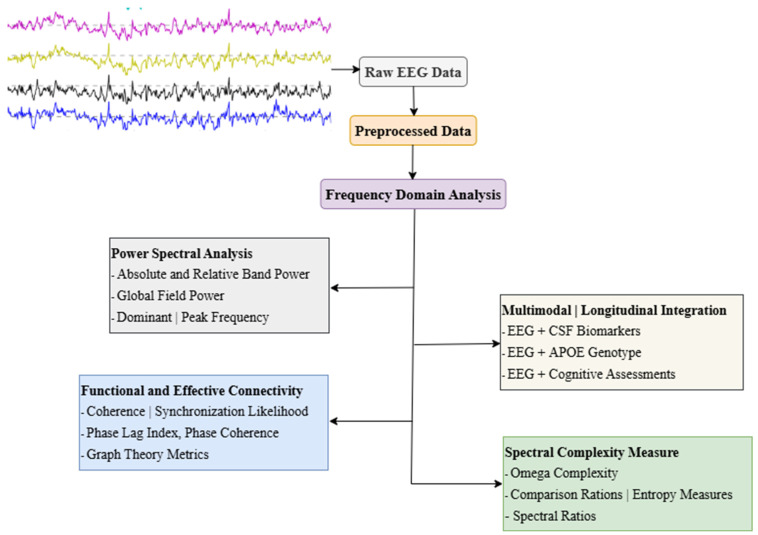
Common frequency-domain EEG analysis approaches in dementia research. Abbreviations: EEG, Electroencephalography; CSF, Cerebrospinal Fluid; APOE, Apolipoprotein E; GFP, Global Field Power; GFS, Global Field Synchronization; PLI, Phase Lag Index. Note: Power Spectral Analysis [5,37,44,47,57]; Functional and Effective Connectivity [40,52,53,56,63]; Spectral Complexity Measures [5,41,42,64]; Multimodal and Longitudinal Integration [46,47,49,58].

In clinical practice, routine resting-state EEG recordings are typically analyzed through visual assessment, which remains essential for examining specific neurophysiological activities. However, frequency-domain analysis of qEEG is required for the objective and practical interpretation of complex EEG data. This approach offers a detailed understanding of EEG signals by breaking them down into frequency components through frequency domain analysis (Figure 1 and Table 1). Furthermore, qEEG generates reliable, quantifiable numerical data, enabling its application in associative and correlative studies for deeper insights [37,38]. It is important to note that qEEG metrics are also influenced by non-pathological factors such as age, sex, and regional background. Healthy aging is commonly associated with normative shifts in EEG rhythms, including reductions in alpha power and elevations in theta activity, while sex-related differences have been reported in global field power (GFP) across multiple frequency bands [39]. These normative variations must be considered when interpreting qEEG alterations to avoid confounding dementia-related findings with normal inter-individual variability.

**Table 1:** Summary of qEEG frequency-domain studies in dementia research.

Study Sample	Approach	Parameters	Reference
12 AD patients, 24 controls	Quantitative EEG	Power spectrum, Omega-complexity, Synchronization likelihood	[5]
40 dementia patients, 15 HCs	Eyes Open/Closed EEG + ML	Spectral features (delta, theta, alpha, beta), ML models)	[27]
AD, DLB, PDD	Advanced qEEG + FOOOF	FFT, AR power (4–8 Hz, 4–15 Hz), dominant/peak frequency, FOOOF model	[28]
205 amyloid+ nondemented (63 SCD, 142 MCI)	Resting-State EEG + Cox Model	Peak frequency, delta, theta, alpha power	[37]
534 (269 HC, 265 AD)	Resting-State qEEG (EO & EC)	PSD, coherence, functional connectivity	[40]
111 (37 AD, 37 MCI, 37 HC)	Resting-State EEG + Cross-Entropy	Cross-ApEn, Cross-SampEn (θ and β1)	[41]
MCI, MiAD, controls	Spectral + Complexity + Synchrony	Relative power, complexity, synchrony (Granger, SES), compression ratios	[42]
44 dementia, 18 MCI, 19 HC	qEEG + Neuropsych Testing	Band powers, clustering analysis	[43]
15 AD treated, 10 untreated	Longitudinal qEEG + AChE Inhibitors	Global Field Power (theta, delta, beta)	[44]
95 SCD (26 amyloid+, 69 amyloid-)	qEEG + Amyloid PET	Delta/alpha1 power, cortical source activity, connectivity	[45]
31 AD (ε4 vs. non-ε4 carriers)	Longitudinal EEG + APOE	Theta/delta activity across APOE groups	[46]
82 (sd-aMCI, md-aMCI)	EEG + CSF biomarkers	GFS, GFP (delta, theta, alpha, beta), CSF tau, neurogranin	[47]
93 (38 AD, 31 MCI, 24 HC)	FFT Dipole Approximation	GFP + source localization (delta–beta)	[48]
637 (SCD, MCI, AD)	EEG + CSF biomarkers	GFP, GFS (delta-beta), CSF Aβ42, p-tau, t-tau	[49]
47 (10 AD, 17 MCI, 20 SCI)	EEG Synchronization Likelihood	SL in beta band (14–22 Hz)	[50]
39 (14 AD, 11 MCI, 14 SCI)	SL (Rest + Working Memory Task)	SL across 0.5–50 Hz, especially alpha/beta	[51]
148 (82 AD, 41 HC, 25 VaD)	SL + Laplacian EEG	Fronto-parietal SL across delta-gamma, focus on alpha1	[52]
266 (69 HC, 88 MCI, 109 AD)	Resting EEG + SL	SL at Fz-Pz, F4-P4; alpha1 + delta bands	[53]
21 AD, 18 controls	Resting-state fMRI + Graph Analysis	Wavelet correlation, Small-world metrics	[54]
15 AD, 13 controls	EEG/MEG Connectivity	PLI, PC, IC, Beta Band	[55]
318 AD, 133 controls	EEG + PLI + MST	PLI, Betweenness Centrality, Hub shift	[56]
22 AD, 23 controls	EEG + GFS	GFS (delta-gamma), MMSE, CDR	[57]
37 AD, 37 controls	EEG + GFS + MoCA/CDR	GFS, MoCA, CDR, K-means clustering	[58]
419 total (HC, MCI, AD)	EEG + GFS	GFS across bands	[59]
19 FTD, 16 AD, 19 controls	qEEG + Neuropsych Testing	GFP, spectral ratio, cognition	[60]
54 AD, 24 Mixed dementia, 66 HC	CT + qEEG + CAMDEX	Slow/fast power, lesion topography	[61]
32 patients (30 dementia), 16 HC	qEEG-SPR + Clinical Assessment	Dementia Index, DLB Index, sensitivity/specificity	[62]

Abbreviations: AD, Alzheimer’s disease; AChE, acetylcholinesterase; AEC-c, amplitude envelope correlation with leakage correction; AR, autoregressive; β1, β2, β3, beta frequency sub-bands 1, 2, and 3; CAMDEX, Cambridge Examination for Mental Disorders of the Elderly; CDR, Clinical Dementia Rating scale; CSF, cerebrospinal fluid; DLB, dementia with Lewy bodies; EEG, electroencephalography; EC, eyes closed; EO, eyes open; FFT, fast Fourier transform; FOOOF, fitting oscillations and one-over-f; FTD, frontotemporal dementia; GFP, global field power; GFS, global field synchronization; HC, healthy controls; ICA, independent component analysis; IC, imaginary component of coherency; LR, logistic regression; MCI, mild cognitive impairment; MiAD, mild Alzheimer’s disease; ML, machine learning; MMSE, Mini-Mental State Examination; MoCA, Montreal Cognitive Assessment; MST, minimum spanning tree; NEUROPSYCH, neuropsychological; PC, phase coherence; PET, positron emission tomography; PLI, phase lag index; PSD, power spectral density; PDD, Parkinson’s disease with dementia; qEEG, quantitative electroencephalography; SCD, subjective cognitive decline; SCI, subjective cognitive impairment; SD-aMCI, single-domain amnestic mild cognitive impairment; MD-aMCI, multi-domain amnestic mild cognitive impairment; SES, stochastic event synchrony; SL, synchronization likelihood; SPR, statistical pattern recognition; VaD, vascular dementia.

## 4. Frequency-Domain Analysis of EEG Changes in AD Spectrum

Frequency-domain analysis is the most widely utilized technique in qEEG for dementia research. It is commonly performed using fast Fourier transform (FFT) spectral and Welch [40,65] analysis (Table 1). The Welch and FFT-based analysis quantifies the amplitude and power of EEG oscillations averaged over a defined recording period across multiple frequency bands, including delta (0.5–4 Hz), theta (4–8 Hz), alpha (8–13 Hz), beta (13–30 Hz), and gamma (30–90 Hz) [40]. These rhythms signify different brain activities, with delta associated with deep sleep and restorative processes, theta linked to memory encoding, emotional regulation, and drowsiness, alpha reflecting relaxation and attentional states, beta indicating active thinking and problem-solving, and gamma representing higher-order cognitive functions such as perception, consciousness, and information integration [66]. The alterations in these rhythms observed in dementia patients provide critical insights into the underlying pathophysiology of cognitive decline and offer potential biomarkers for disease progression and therapeutic monitoring [36,37,38,39,40,65,66,67].

The generation of characteristic cortical rhythms, traditionally categorized into slow (delta and theta) and fast (alpha, beta, and gamma) frequency activity, has been extensively debated, with thalamocortical circuits and cortical regions identified as the primary contributors to these patterns [68,69,70]. Numerous qEEG studies have demonstrated increased amplitude and power in slow-frequency bands, commonly referred to as generalized EEG slowing, in individuals along the clinical AD spectrum [38,40,42]. These alterations have been shown to correlate with and predict neuropsychological deficits, including impairments in memory, attention, verbal skills, frontal lobe function, and daily living activities in cognitively impaired individuals [43,67]. Furthermore, these changes appear to follow a progressive trajectory, characterized by an initial increase in theta power and a reduction in beta power, followed by a decline in alpha power, and ultimately a late-stage increase in delta power, typically observed in the more advanced stages of AD [38,42].

The earliest qEEG markers typically emerge during SCD and early MCI, with a notable increase in frontal theta power and reduction in posterior alpha activity [71]. These electrophysiological changes often precede abnormalities in CSF or PET markers, reflecting early synaptic disconnection rather than irreversible neuronal loss [18]. This pattern supports the utility of qEEG as a sensitive tool for detecting prodromal alterations in neural activity and cognitive processing. In contrast, increased delta power and reduced GFS tend to be more pronounced in moderate-to-late-stage AD, offering a temporal gradient of qEEG marker evolution across the disease continuum [18,47]. These stage-specific electrophysiological signatures reinforce the potential of frequency-domain qEEG analysis in tracking disease progression and differentiating early from late neuropathological changes.

Longitudinal qEEG investigations have revealed a temporally dynamic trajectory in electrophysiological biomarkers, with one prospective study documenting significant longitudinal deterioration in quantitative EEG parameters among AD patients over a 12-month observation period compared to baseline metrics [44,45,46]. Complementing this, Simfukwe et al. demonstrated that comparative analysis of eyes-open versus eyes-closed resting-state EEG configurations employing Welch spectral estimation identified beta and alpha frequency modulations as the most robust electrophysiological predictors of incipient cognitive deterioration in AD cohorts [40]. Diverging from conventional FFT-derived power spectral profiling, emerging research paradigms have prioritized global field power (GFP), a spatially integrative metric calculated as the spatial standard deviation of scalp potentials across all electrode pairs, reflecting whole-brain field strength dynamics [47]. Huang et al. reported marked elevations in delta and theta GFP coupled with attenuated alpha GFP in AD patients relative to cognitively intact controls, alongside significant attenuation of alpha and beta GFP amplitudes in AD compared to mild MCI cohorts [48]. Notably, their work further identified diminished alpha GFP in progressive MCI converters relative to non-converter counterparts, suggesting its potential as a prognostic indicator of neurodegenerative progression [48]. Robust intergroup disparities in delta, theta, and alpha GFP spectral profiles were observed within a multicenter cohort exceeding 600 individuals across the AD continuum, encompassing subjective cognitive decline (SCD), MCI, and AD [49]. These results demonstrate the methodological validity of reference-independent GFP-derived metrics, affirming their reproducibility and analytical congruence with established multichannel FFT-derived spectral power assessments.

The diffuse neuropathological burden of AD, marked by progressive neuronal and synaptic degeneration culminating in pan-cognitive functional deterioration, has motivated rigorous investigation into the dysregulation of large-scale neural circuit synchronization across the AD continuum. EEG coherence has emerged as a predominant analytical for probing aberrant functional connectivity and neurophysiological network disorganization [50,72,73]. Coherence quantifies phase-locked oscillatory coupling between paired EEG signals derived from spatially distinct scalp electrodes within defined spectral bands [73,74]. Empirical evidence has consistently demonstrated global attenuation of interregional coherence across all frequency bands in AD cohorts, with deficits most pronounced within the alpha (8–12 Hz) frequency range [75,76,77].

Despite its utility, EEG coherence is limited by the volume conduction effect, which occurs due to the spreading of electrical currents through biological tissues. This phenomenon can artificially inflate measures of interdependence between channels, leading to potential overestimations of functional connectivity [73,78]. Stam et al. [63] proposed Synchronization Likelihood (SL), an advanced nonlinear metric designed to quantify non-stationary, nonlinear interdependencies among multichannel EEG signals. SL has been validated to exhibit robust correlations with global cognitive performance metrics, demonstrating progressive attenuation across all spectral bands (delta to gamma) in neurodegenerative cohorts, including MCI and AD, relative to neurotypical controls in both cross-sectional and longitudinal analyses.

In addition to coherence and SL, topographic EEG mapping offers a valuable tool for examining brain connectivity by providing spatial visualizations of neural activity across the scalp for various frequency bands [51,52,53,79]. These maps give researchers insight into specific disruptions in brain networks and allow them to identify region-specific abnormalities and connection patterns in AD patients. As an illustration, topographic mapping research has revealed notable decreases in functional connectivity in the frontal and parietal areas, specifically in the alpha and beta frequency bands, essential for cognitive functions like executive function, memory, and attention [80,81]. Simfukwe et al. investigated topographic EEG mapping to investigate functional connectivity differences between AD patients and healthy controls. Their analysis focused on the relative power spectral density (PSD) from frequency-domain analysis qEEG in the beta frequency range, revealing distinct connectivity patterns between the two groups. Topographic maps highlighted more localized functional connections in both groups at electrodes with high PSD values, including Fp2, T4, Cz, F7, Pz, O2, F4, and C4 [40]. However, in the AD group, topographic mapping showed significantly fewer strong connections between frontal and temporal regions compared to the healthy control group. This reduction in connectivity indicates diminished synchronization and impaired information transfer in the AD group, reflecting the disruption of neural networks critical for cognitive function [54,82].

The Phase Lag Index (PLI) has emerged as an innovative methodological framework for assessing EEG-based neural synchrony, circumventing confounding biases introduced by volume conduction artifacts that plague traditional coherence metrics [55]. Empirical investigations have established that AD cohorts manifest attenuated PLI values, specifically within the alpha (8–12 Hz) and beta (13–30 Hz) spectral bands, exhibiting strong clinicopathological correlations with advancing disease severity [55,56]. A critical methodological constraint of PLI lies in its bivariate analytical, which restricts interpretation to pairwise signal interactions and neglects higher-order network dynamics critical for large-scale neural integration. To overcome this fundamental constraint, Koenig et al. pioneered an integrative analytical paradigm that distills multichannel EEG data into a unified global connectivity index across spectral domains. This approach, termed Global Field Synchronization (GFS), operates as a reference-independent metric to quantify instantaneous phase coherence across spatially distributed neural populations, providing a spatially comprehensive assessment of whole-brain network integration. Unlike PLI, GFS considers all electrodes simultaneously and does not rely on assumptions regarding the location of active sources, offering a holistic view of brain connectivity by including all oscillatory activity co-occurring [83].

Additionally, findings from Park et al. and Ma et al. [57,58] have demonstrated that perturbations in GFS exhibit robust clinical-electrophysiological correlations with cognitive performance in AD cohorts, as operationalized through standardized neuropsychological instruments, including the Mini-Mental State Examination (MMSE), Montreal Cognitive Assessment (MoCA), and Clinical Dementia Rating (CDR) Scale. These observations substantiate GFS as a multidimensional biomarker capable of concurrently capturing aberrant large-scale network integration and cognitive decline in AD. Furthermore, pan-stage dysregulation of neurophysiological network integrity across the AD continuum spanning preclinical to clinical phases has been systematically characterized via qEEG frequency-domain metrics interrogating functional connectivity architecture. This body of evidence consolidates the translational utility of qEEG spectral decomposition as a neurophysiological lens for probing AD-associated network pathophysiology and disease progression [40,59].

## 5. Frequency-Domain Analysis in the Differential Diagnosis of Dementia

Recent findings indicate that a significant proportion of clinically diagnosed AD cases exhibit neurodegenerative heterogeneity, with many individuals phenotypically aligned with AD presenting mixed neuropathological substrates or novel dementia subtypes, some biologically characterized only in contemporary neuropathological [84,85,86,87]. Although qEEG has shown strong potential in capturing neural signatures associated with dementia, its ability to reliably distinguish AD from other neurological conditions remains limited. Spectral slowing characterized by elevated delta and theta power and reduced alpha and beta activity is not exclusive to AD and is also observed in disorders such as traumatic brain injury, metabolic encephalopathy, and major depressive disorder [88,89,90]. This overlap complicates differential diagnosis in clinical settings. To address this, future studies should aim to identify disorder-specific qEEG markers or integrate qEEG with complementary modalities such as structural MRI, PET imaging, and comprehensive neuropsychological assessments. In addition, advanced analytic approaches, including machine learning (ML) models trained on multimodal data, hold promise for improving the specificity of qEEG and enabling more accurate patient-level classification. Ultimately, ideal biomarkers for AD must align temporally with clinical progression while also exhibiting molecular specificity to differentiate AD’s hallmark pathologies, such as amyloid-β plaques and tau tangles, from other coexisting or confounding neurological conditions.

The EEG signals display marked interindividual heterogeneity in oscillatory dynamics [87,91], highlighting the necessity of quantitative computational frameworks to mitigate subjective interpretive biases and enable objective neurophysiological phenotyping. Notably, EEG signal variability, a putative marker of neurophysiological dysregulation and network rigidity, has garnered increasing attention as a novel dimension of neural dysfunction in neurodegenerative disease research [64,92,93]. In a seminal investigation, Lehmann et al. established that multivariate qEEG signatures integrating amplitude, spectral power, and synchronization indices achieved discriminative validity with sensitivities of 85% (mild AD) and 89% (moderate AD), alongside specificities of 78% and 88%, respectively, when distinguishing AD cohorts from neurotypical controls [60]. These findings are corroborated by a growing body of evidence demonstrating robust cross-cohort classification for qEEG-derived biomarkers in differentiating MCI and AD from healthy aging [27,33,40,66,94].

In the context of neurophysiological differentiation between AD and frontotemporal dementia (FTD), this has been elucidated through EEG biomarkers. Lindau et al. established that FTD cohorts display a marked attenuation of fast-frequency (beta/gamma) spectral power, in stark contrast to AD patients, who exhibit pathognomonic elevations in slow-frequency (delta/theta) oscillatory activity, a divergence underscoring disease-specific spectral signatures [61]. Furthermore, FTD patients exhibit significantly diminished global synchronization across all frequency bands relative to healthy controls and AD cohorts, underscoring the diagnostic GFS in disentangling neurodegenerative etiologies [61]. Source-localized EEG analyses have identified distinct spatial signatures of oscillatory dysregulation: FTD is characterized by attenuated alpha 1 (8–10 Hz) activity localized to orbitofrontal and temporal cortices, whereas AD manifests diffuse delta (1–4 Hz) hyperactivity alongside pronounced beta 1 (13–20 Hz) suppression in parietal and sensorimotor regions, with topographical patterns reflecting pathophysiological divergence between these dementias [95]. These findings collectively underscore the translational potential of multimodal EEG metrics, integrating spectral, synchronization, and source-space analyses to refine differential diagnosis and elucidate network-level pathophysiology in neurodegenerative disorders.

In contrast, the neurophysiological divergence between dementia subtypes is further exemplified in mixed dementia (MD), characterized by comorbid vascular and Alzheimer’s pathologies. qEEG analyses reveal that MD exhibits a unique PSD pattern marked by a pronounced elevation in slow-frequency (delta/theta) oscillatory power, contrasting with the canonical fast-frequency (beta) attenuation observed in pure AD, a pattern divergent from the FTD phenotype [62]. Among non-AD dementias, dementia with Lewy bodies (DLB) represents the second most prevalent etiology [96]. Pathognomonic EEG signatures in DLB include posterior-dominant rhythmic slowing (PDRS), with delta/theta power increases in occipital-parietal regions exceeding the magnitude observed across the AD continuum [96,97,98]. These disease-specific oscillatory signatures emerge during prodromal stages and exhibit robust temporal correlations with cognitive fluctuation severity, a hallmark clinical feature of DLB [97]. These findings underscore the discriminative capacity of spectral biomarkers in disentangling complex dementia etiologies and staging disease progression.

Additionally, the evidence further highlights the dysregulation of resting-state network architecture and large-scale network dynamics in DLB, characterized by attenuated EEG coherence and pathological reconfiguration of functional connectivity profiles across cortico-subcortical circuits [98,99,100]. In alignment with these findings, the revised diagnostic criteria issued by the Dementia with Lewy Bodies Consortium now formally endorse EEG as an ancillary diagnostic biomarker for DLB [96], driven by its demonstrated capacity to resolve clinicopathological ambiguity through spectral and network-based signatures that demonstrate superior discriminative validity in etiological differentiation particularly distinguishing DLB from AD and other neurodegenerative dementias [101,102,103,104]. Most studies investigating the classification potential of frequency domain analysis in dementia have focused on group-level analyses. Future validation studies should focus on assessing and validating qEEG’s efficacy as a diagnostic tool at the individual subject level, as this is crucial for clinical practice acceptance.

## 6. ML Approaches for Identifying qEEG Biomarkers in Dementia Research

Recent advances in ML have significantly enhanced the capacity of qEEG analysis to identify diagnostic and prognostic biomarkers for AD and related dementias. Traditional supervised ML algorithms, including support vector machines (SVMs), random forests (RF), and k-nearest neighbors (KNN), have shown moderate to high classification performance when applied to qEEG features such as power spectral densities, coherence, and entropy-based metrics [18,105]. More recent efforts have shifted toward deep learning (DL) techniques, particularly convolutional neural networks (CNNs) and long short-term memory (LSTM) architectures, which can learn complex, hierarchical patterns directly from raw EEG signals or time-frequency representations like spectrograms and scalograms [106,107,108]. These models not only improve diagnostic accuracy but also show potential for early-stage dementia detection and progression forecasting in longitudinal studies.

## 7. Conclusions

Time-frequency domain analysis of qEEG has shown substantial promise in uncovering neural alterations associated with dementia, particularly AD. Across studies, spectral features such as delta, theta, alpha, and beta band powers, combined with advanced metrics including GFS, GFP, SL, complexity measures (Omega-complexity, Cross-ApEn, Cross-SampEn), and entropy-based connectivity indices, have shown meaningful associations with cognitive decline, disease severity, and biological markers like CSF tau, Aβ42, and neurogranin. These neurophysiological changes reflect underlying synaptic dysfunction and may precede structural damage, supporting the value of qEEG for early detection.

Despite these advantages, clinical translation remains limited by inconsistent validation against established biomarkers and heterogeneity in analytic methodologies. A further challenge is the absence of standardized EEG preprocessing pipelines, which impedes reproducibility and cross-study comparisons. Moreover, many of the reviewed studies did not adequately account for confounding variables such as age, sex, APOE genotype, regional background, and medication use. This highlights the need for multivariate statistical models that can isolate the independent predictive value of qEEG features in dementia biomarker research.

Emerging analytic approaches such as FOOOF offer new opportunities to dissect oscillatory components more precisely, but their comparative value over traditional band-power methods remains insufficiently explored. Future investigations should apply these methods side-by-side within the same datasets to better assess diagnostic performance. Multimodal integration linking time-frequency domain qEEG with neuroimaging (MRI, PET), CSF biomarkers, and longitudinal clinical data will be essential for mapping the temporal trajectory of dementia and enhancing diagnostic precision.

This review contributes uniquely by consolidating underutilized qEEG metrics across dementia subtypes, identifying methodological gaps, and advancing a research agenda for standardization and multimodal synthesis. Notably, qEEG holds particular promise for detecting early synaptic dysfunction in SCD and MCI, where early changes in frontal theta, posterior alpha, and network connectivity may precede structural or biochemical abnormalities. These insights support its potential for scalable, non-invasive screening in preclinical stages.

Given the overlap of spectral features between dementia and other neurological conditions such as traumatic brain injury and major depressive disorder, future research should aim to refine diagnostic specificity by identifying disorder-specific electrophysiological signatures and incorporating complementary modalities. This includes the integration of qEEG with structural and molecular imaging, neuropsychological profiling, and ML approaches.

While ML has enhanced the potential of qEEG-based diagnostics, its clinical application is still constrained by challenges such as interpretability, limited generalizability, and the need for standardized input features. Future studies should prioritize the development of explainable DL frameworks and validate their performance across multicenter cohorts, particularly through integration with imaging and fluid biomarkers to improve reliability and clinical utility. Overall, these directions offer a roadmap for advancing qEEG as a standardized, multimodal biomarker platform for the early diagnosis and monitoring of dementia.
